# The impact of hospital safety-net status on inpatient outcomes for brain tumor craniotomy: a 10-year nationwide analysis

**DOI:** 10.1093/noajnl/vdaa167

**Published:** 2020-12-01

**Authors:** Oliver Y Tang, Krissia M Rivera Perla, Rachel K Lim, Robert J Weil, Steven A Toms

**Affiliations:** 1 Department of Neurosurgery, The Warren Alpert Medical School of Brown University, Providence, Rhode Island, USA; 2 Department of Neurosurgery, Rhode Island Hospital, Providence, Rhode Island, USA

**Keywords:** glioma, meningioma, metastasis, social determinants of health, vestibular schwannoma

## Abstract

**Background:**

Outcome disparities have been documented at safety-net hospitals (SNHs), which disproportionately serve vulnerable patient populations. Using a nationwide retrospective cohort, we assessed inpatient outcomes following brain tumor craniotomy at SNHs in the United States.

**Methods:**

We identified all craniotomy procedures in the National Inpatient Sample from 2002–2011 for brain tumors: glioma, metastasis, meningioma, and vestibular schwannoma. Safety-net burden was calculated as the number of Medicaid plus uninsured admissions divided by total admissions. Hospitals in the top quartile of burden were defined as SNHs. The association between SNH status and in-hospital mortality, discharge disposition, complications, hospital-acquired conditions (HACs), length of stay (LOS), and costs were assessed. Multivariate regression adjusted for patient, hospital, and severity characteristics.

**Results:**

304,719 admissions were analyzed. The most common subtype was glioma (43.8%). Of 1,206 unique hospitals, 242 were SNHs. SNH admissions were more likely to be non-white (*P* < .001), low income (*P* < .001), and have higher severity scores (*P* = .034). Mortality rates were higher at SNHs for metastasis admissions (odds ratio [OR] = 1.48, *P* = .025), and SNHs had higher complication rates for meningioma (OR = 1.34, *P* = .003) and all tumor types combined (OR = 1.17, *P* = .034). However, there were no differences at SNHs for discharge disposition or HACs. LOS and hospital costs were elevated at SNHs for all subtypes, culminating in a 10% and 9% increase in LOS and costs for the overall population, respectively (all *P* < .001).

**Conclusions:**

SNHs demonstrated poorer inpatient outcomes for brain tumor craniotomy. Further analyses of the differences observed and potential interventions to ameliorate interhospital disparities are warranted.

**Key Points**

- Safety-net hospital (SNH) brain tumor patients had higher presentation severity.
- SNHs were more likely to be government-owned and have lower brain tumor volumes.
- Inpatient complications, length of stay, and costs were elevated at SNHs.

**Importance of the Study**

Several studies have demonstrated poorer outcomes at safety-net hospitals (SNHs), which disproportionately serve Medicaid and uninsured patients. However, the only nationwide study that has characterized brain tumor SNH outcomes focused exclusively on glioblastoma, and certain outcomes like costs are poorly understood. This study assessed a nationally generalizable population of 304,719 admissions for brain tumor craniotomy for glioma, metastasis, meningioma, and vestibular schwannoma (VS). We documented notable interhospital disparities in the surgical management of brain tumors, including higher mortality for metastasis, increased complications for meningioma, and elevated length of stay and costs for all four subtypes at SNHs. This is the first study to characterize SNH outcomes for meningioma and VS, and the first nationwide assessment of SNH outcomes for non-glioma brain tumors. This is a timely topic given the stresses on SNHs due to the coronavirus pandemic and crucial discussions about social disparities taking place in the United States and worldwide.

The treatment of brain tumors is a resource-intensive and long-term care process. Factors outside the formal health care setting like social determinants of health (SDoH)—the political, socioeconomic, and environmental factors that shape health access, care, and outcomes—may influence short- and long-term outcomes.^1^ Accordingly, earlier studies demonstrated that incidence, operative outcomes, and long-term survival for brain tumors may be influenced by SDoH, such as a patient’s race and ethnicity, insurance type, and socioeconomic status.^2–5^ Safety-net hospitals (SNHs), which serve elevated numbers of vulnerable patients, including those with Medicaid coverage and the uninsured, face additional challenges. While comprising only a quarter of all hospitals nationally, SNHs treat over half of Medicaid and uninsured patients.^6^ Prior research has documented poorer outcomes at SNHs due to resource limitations and advanced disease at presentation among patients.^7,8^ Continued cuts to Disproportionate Share Hospital payments subsidizing SNHs place hundreds of SNHs at risk of closure.^9^

It has been suggested that SNHs struggle most with technically demanding procedures and subspecialty care.^10^ While several single-institution studies have documented poorer outcomes for brain tumor admissions treated at SNHs,^11–14^ certain outcomes like hospital costs are less characterized. Moreover, the only nationwide analysis of SNH brain tumor treatment to date focused exclusively on glioblastoma.^11^ Using a nationally generalizable cohort, we examined the association between hospital safety-net status and perioperative outcomes for craniotomy patients in four different brain tumor subtypes.

## Materials and Methods

### Data Source and Inclusion Criteria

We analyzed the National Inpatient Sample (NIS), the largest all-payer inpatient database in the United States.^15^ Curated by the Healthcare Cost and Utilization Project (HCUP), NIS contains a 20% stratified sample of all U.S. nonfederal hospital discharges and reports patient, hospital, and severity variables for approximately 7 million admissions annually. Using previously validated criteria and International Classification of Diseases, 9th Edition (ICD-9) diagnosis and procedure codes,^2,16^ we identified all adult admissions (≥18 years old) from 2002 to 2011 undergoing a craniotomy for one of four tumor subtypes: glioma, metastasis, meningioma, or vestibular schwannoma (VS; Table 1). Patients undergoing craniotomies for multiple tumor subtypes in a single inpatient stay (<1%) were excluded. The NIS stopped including all admissions for individual hospitals after 2012, thus making accurate calculations of safety-net burden beyond 2012 unfeasible. Due to the anonymized nature of the NIS, this study was exempt from Institutional Review Board review.

**Table 1.** Selection Criteria for Brain Tumor Admission Subtypes

Brain Tumor Subtype	Inclusion Criteria	Subtype-Specific Severity Metrics
Glioma	*Diagnosis:* 191.0–191.5, 191.8, 191.9, 225.0, 237.5	*Performance of resection:* 01.53, 01.59
	*Procedure:* 01.13, 01.14, 01.53, 01.59	*Malignant status and location within brain:* 191.0–191.5, 191.8, 191.9
Metastasis	*Diagnosis:* 198.3	*Presence of other extracranial metastasis:* 197.0–197.8, 198.0–198.2, 198.5–198.7, 198.81, 198.82, 198.89, 199.0
	*Procedure:* 01.59	*Lung cancer:* 162.0–9
Meningioma	*Diagnosis:* 225.2, 192.1, 237.6	*Malignant status:* 191.1
	*Procedure:* 01.51	
Vestibular Schwannoma	*Diagnosis:* 225.1	*Hydrocephalus:* 331.3, 331.4
	*Procedure:* 04.01	*Neurofibromatosis diagnosis:* 237.7, 237.70–237.72

Inclusion criteria were based off of ICD-9 diagnosis and procedure codes. For subtype analysis, each subtype had different severity metrics that were included as confounders in multivariate regression.

Because >20% admissions did not report race, we utilized multinomial logistical regression to impute missing race data following HCUP’s methodology.^17^ Admissions with remaining missing data were excluded from multivariate analysis (Supplementary Figure 1). ICD-9 diagnosis codes were used to code tumor-specific severity metrics using previously-reported methods (Table 1).^2^ For glioma, we identified performance of a resection (compared to biopsy), histopathological determination (malignant or benign), and lesion location. For metastasis, we identified the presence of extracranial metastases and diagnosis of lung cancer. For meningioma, we identified malignant status. For VS, we identified diagnosis of neurofibromatosis and hydrocephalus at presentation.

### Classification of Hospital Characteristics

Following Trinh et al., we classified hospitals in the top quartile of brain tumor caseload as “high-volume hospitals,” and the remaining hospitals as “low-volume hospitals.” ^16^ We calculated safety-net burden for each hospital as the number of included admissions covered by Medicaid or uninsured divided by total admissions over 2002–2011.^8,10,18–20^ Hospitals in the top quartile of safety-net burden were defined as SNHs; all others were “non-safety-net hospitals.” ^8,10,18–20^ This was our study’s primary independent variable.

### Outcomes at SNHs

Outcomes of interest included inpatient mortality, favorable discharge disposition, complications, hospital-acquired conditions (HAC), overall length of stay (LOS), postoperative LOS, hospital costs, favorable discharge disposition, and inpatient mortality. Disposition was dichotomized into favorable vs. unfavorable following the methodology of Clement et al., with discharge to home or short-term hospital classified as a favorable outcome.^21^ Complications were identified utilizing Clinical Classifications Software groupings, which identify the most common inpatient medical and surgical complications, and ICD-9 codes from earlier neurosurgical NIS studies (Supplementary Table 1).^22–24^ HACs, tracked by the Center for Medicare & Medicaid Services (CMS) and used to determine reimbursement rates, are high-cost and high-volume, preventable complications used to quantify quality of care. HACs were identified using CMS-defined ICD-9 codes as outlined in Lopez Ramos et al.^8^ Inpatient costs were estimated by multiplying reported inpatient charges by all-payer hospital-specific cost-to-charge ratios provided by the CMS.^13^

### Statistical Analysis

Using Stata 15 (StataCorp) and *svy* commands, we applied survey weights to make national estimates. Nonparametric Mann–Whitney and Kruskal–Wallis tests identified differences in characteristics and unadjusted outcomes between patients at SNHs and non-SNHs. Multivariate regression was used to adjust for 13 confounding variables: patient demographics (age, sex, race, insurance status, and income quartile of ZIP code), general severity metrics (All Patient Refined Diagnosis Related Group [APR-DRG] severity of illness and risk of mortality scores, Charlson Comorbidity Index, admission type), and hospital characteristics (ownership, location and teaching status, Census region, and high-volume status). Unique multivariate models were constructed for each of the four brain tumor subtypes including subtype-specific severity metrics as additional confounders.

For binary variables, we performed logistic regression and reported odds ratios (ORs). For LOS and inpatient costs, we performed gamma regression with a log-link function, idealized for modeling continuous right-skewed outcomes, and reported β-coefficients. β-coefficients correspond to the percent change in the outcome (ex. β-coefficient = 1.06 indicates 6% increase).

We used random-effects meta-analyses to combine separate outcomes for each subtype into a single “pooled outcome” representing the entire study population.^2^ We calculated Cochran’s Q statistic for each pooled outcome to rule out significant study heterogeneity, defined as *P* < .10. Statistical significance was maintained at *P* < .05.

## Results

### Nationwide Brain Tumor Craniotomy Admission Characteristics

**Figure 1.**
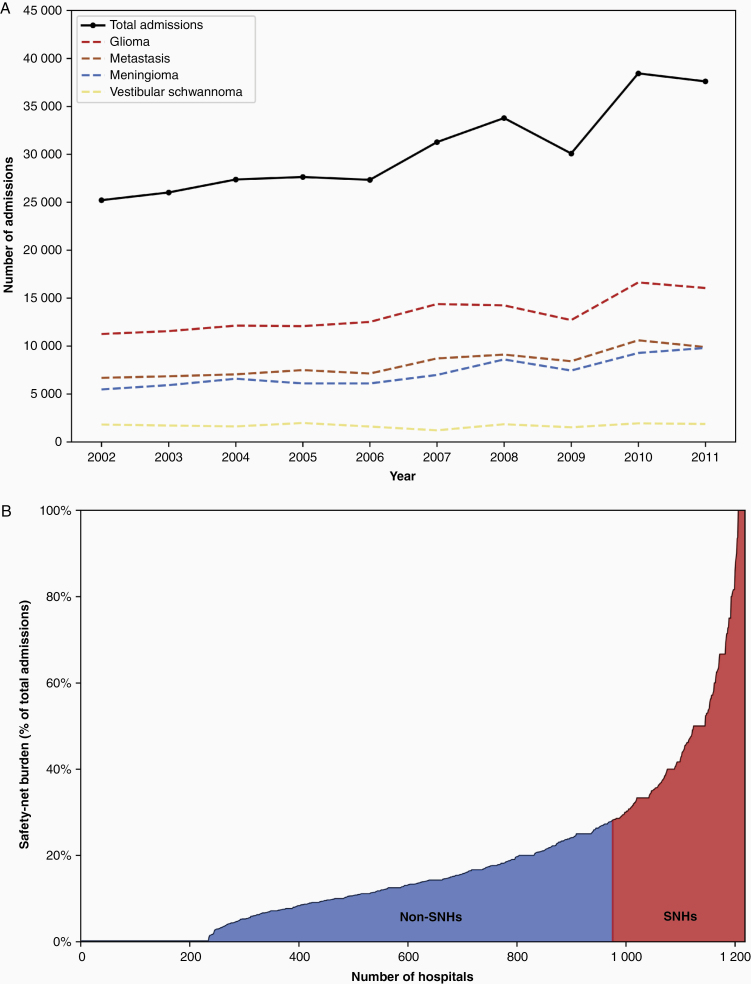
Nationwide admissions for brain tumor craniotomy. (A) Number of admissions from 2002 to 2011 for craniotomy for all brain tumors and each subtype. (B) Cumulative plot of all 1,206 hospitals in the study population, in ascending order by their respective safety-net burden (y-axis). Safety-net burden was quantified as the number of Medicaid and uninsured admissions divided by total admissions. Two hundred and forty-two hospitals were classified as safety-net hospitals, with the threshold for classification being a safety-net burden of ≥28.6%.

A total of 304,719 nationwide admissions for brain tumor craniotomy from 2002 to 2011 were analyzed, after exclusions (Supplementary Figure 1). The most common tumor subtype was glioma (43.8%; Figure 1A). The average age was 56.1 years old (standard deviation [SD] = 14.8), and most patients were female (52.4%) and white (80.3%; Table 2). Patients predominantly had private insurance (52.2%), while Medicaid and uninsured patients comprised 15.9% of admissions. The majority of patients were treated at a private nonprofit (76.0%), urban teaching (77.9%), and high-volume hospital (71.4%).

**Table 2.** Characteristics for Nationwide Brain Tumor Craniotomy Admissions from 2002 to 2011

Characteristics	Total Number (%)
Total admissions	304,719 (100.0)
Tumor subtypes	
Glioma	133,505 (43.8)
Metastasis	81,888 (26.9)
Meningioma	72,260 (23.7)
Vestibular Schwannoma	17,066 (5.6)
Age (years): Mean ± SD	56.1 ± 14.8
IQR	47–67
Sex	
Male	145,134 (47.6)
Female	159,585 (52.4)
Race	
White	244,596 (80.3)
Black	24,924 (8.2)
Hispanic	20,312 (6.7)
Asian or Pacific Islander	5,264 (1.7)
Native American	1,255 (0.4)
Other	8,366 (2.8)
Insurance status	
Medicaid or uninsured	48,400 (15.9)
Medicare	97,248 (31.9)
Private insurance	159,071 (52.2)
Income quartile of patient’s ZIP Code	
0–25% (Lowest)	63,082 (20.7)
25–50%	74,580 (24.5)
50–75%	78,554 (25.8)
75–100% (Highest)	88,503 (29.0)
APR-DRG severity of illness	
1 (Minor)	98,014 (32.2)
2 (Moderate)	135,524 (44.5)
3 (Major)	52,809 (17.3)
4 (Extreme)	18,372 (6.0)
APR-DRG risk of mortality	
1 (Minor)	163,598 (53.7)
2 (Moderate)	84,447 (27.7)
3 (Major)	35,705 (11.7)
4 (Extreme)	20,969 (6.9)
Charlson Comorbidity Index: Mean ± SD	3.3 ± 3.2
IQR	1–6
Admission Type	
Emergency	77,088 (25.3)
Urgent	46,164 (15.2)
Elective	181,467 (59.6)
Hospital ownership	
Government	53,030 (17.4)
Private non-profit	231,562 (76.0)
Private for-profit	20,127 (6.6)
Hospital location and teaching status	
Rural	7,715 (2.5)
Urban non-teaching	59,723 (19.6)
Urban teaching	237,281 (77.9)
Hospital census region	
Northeast	67,007 (22.0)
Midwest	72,782 (23.9)
South	127,210 (41.8)
West	37,720 (12.4)
Hospital volume status	
Low volume	87,061 (28.6)
High volume	217,658 (71.4)
Hospital safety-net status	
SNH	269,247 (88.4)
Non-SNH	35,472 (11.6)

Percentages may not add up to 100% due to rounding.

APR-DRG, All patients refined diagnosis-related groups.

### SNH Characteristics

Of the 1,206 unique hospitals in the study population, 242 hospitals (20.1%) were classified by safety-net burden as SNHs. The top quartile of safety-net burden included hospitals with ≥28.2% Medicaid or uninsured admissions (Figure 1B). SNHs were more likely to be low-volume (83.9% vs 75.3%, *P* = .005) and government-owned (25.2% vs 6.8%, *P* < .001).

Of total admissions, 35,472 (11.6%) received a brain tumor craniotomy at an SNH. Compared to their non-SNH counterparts, patients at SNHs were younger (mean age 54.0 vs 56.4, *P* < .001), non-white (38.4% vs 17.3%, *P* < .001), and from ZIP codes in the bottom income quartile (35.6% vs 18.7%, *P* < .001; Table 3). Insurance source was significantly different between SNH and non-SNH admissions (*P* < .001), with a majority of non-SNH patients having private insurance (54.8%) and a plurality of SNH patients enrolled in Medicaid or uninsured (40.7%). There was a significantly higher proportion of meningioma, but lower proportion of glioma and vestibular schwannoma admissions at SNHs (*P* < .001). SNH patients more often had nonelective admission (47.1% vs 39.6%, *P* < .001) and had higher severity of illness (mean 2.0 vs 1.9, *P* = .034) and risk of mortality scores (mean 1.8 vs 1.7, *P* = .021). Glioma admissions at SNHs were less likely to receive resection instead of just open biopsy (93.3% vs 94.2%, *P* = .019). Metastasis admissions at SNHs were more likely to have a lung cancer diagnosis (38.1% vs 33.4% *P* < .001). An analysis of other common primary tumor sites for brain metastasis determined that diagnoses of breast cancer (5.5% vs 3.4%, *P* < .001) and colon cancer (1.4% vs 1.1%, *P* = .044) were also significantly higher among SNH patients, but there were no differences for skin or kidney cancer (Supplementary Table 2).^25^ There were no differences in extracranial metastases. Finally, SNH VS admissions more frequently presented with hydrocephalus (8.3% vs 4.3%, *P* < .001).

**Table 3.** Differences in Patient Demographics, General Severity Metrics, and Subtype-Specific Severity Metrics at SNHs and Non-SNHs

Subtype and Severity Metrics	Total Number (%) for Non-SNHs	Total Number (%) for SNHs	*P* Value
Tumor subtypes			.009***
Glioma	118,791 (44.1%)	14,713 (41.5%)	
Metastasis	72,370 (26.9%)	9,518 (26.8%)	
Meningioma	62,622 (23.3%)	9,638 (27.2%)	
Vestibular Schwannoma	15,464 (5.7%)	1,602 (4.5%)	
Age (years): Mean ± SD	54.0 ± 14.8	56.4 ± 14.8	<.001***
Race			<.001***
White	222,731 (82.7%)	21,865 (61.6%)	
Black	20,046 (7.4%)	4,878 (13.8%)	
Hispanic	14,092 (5.2%)	6,220 (17.5%)	
Asian or Pacific Islander	4,530 (1.7%)	734 (2.1%)	
Native American	984 (0.4%)	271 (0.1%)	
Other	6,862 (2.5%)	1,504 (4.2%)	
Insurance status			<.001***
Medicaid or uninsured	33,964 (12.6%)	14,436 (40.7%)	
Medicare	87,829 (32.6%)	9,419 (26.6%)	
Private insurance	147,455 (54.8%)	11,616 (32.7%)	
Income Quartile of Patient’s ZIP Code			.001***
0–25% (Lowest)	50,464 (18.7%)	12,618 (35.6%)	
25–50%	64,862 (24.1%)	9,718 (27.4%)	
50–75%	70,721 (26.3%)	7,833 (22.1%)	
75–100% (Highest)	83,200 (30.9%)	5,303 (15.0%)	
APR-DRG severity of illness			.034***
1 (Minor)	86,549 (32.1%)	11,466 (32.3%)	
2 (Moderate)	120,010 (44.6%)	15,514 (43.7%)	
3 (Major)	46,708 (17.3%)	6,100 (17.2%)	
4 (Extreme)	15,980 (5.9%)	2,392 (6.7%)	
APR-DRG risk of mortality			.021***
1 (Minor)	144,590 (53.7%)	19,007 (53.6%)	
2 (Moderate)	74,580 (27.7%)	9,867 (27.8%)	
3 (Major)	31,589 (11.7%)	4,116 (11.6%)	
4 (Extreme)	18,488 (6.9%)	2,482 (7.0%)	
Charlson Comorbidity Index: Mean ± SD	3.4 ± 0.01	3.4 ± 0.01	.136
Admission type			<.001***
Emergency	64,847 (24.1%)	12,241 (34.5%)	
Urgent	41,683 (15.5%)	4,481 (12.6%)	
Elective	162,717 (60.4%)	18,750 (52.9%)	
Glioma: Performance of resection	111,862 (94.2%)	13,725 (93.3%)	.019***
Glioma: Malignant status	104,103 (87.6%)	12,081 (82.1%)	<.001***
Metastasis: Presence of other extracranial metastasis	17,457 (24.1%)	2,259 (20.9%)	.926
Metastasis: Lung cancer	24,157 (33.4%)	3,625 (38.1%)	<.001***
Meningioma: Malignant status	2,480 (4.1%)	317 (3.3%)	.204
Vestibular Schwannoma: Hydrocephalus	663 (4.3%)	133 (8.3%)	<.001***
Vestibular Schwannoma: Neurofibromatosis diagnosis	255 (1.6%)	39 (2.4%)	.285

Percentages may not add up to 100% due to rounding. Nonparametric Mann–Whitney and Kruskal–Wallis tests were used to detect significant differences in characteristics between admissions at SNHs compared to admissions at non-SNHs. Subtype-specific severity metrics were listed following their relevant subtype (eg, “Glioma: Performance of resection”).

APR-DRG, All patients refined diagnosis-related groups.

(***) denotes statistical significance.

When comparing unadjusted inpatient outcomes using univariate logistic regression or Mann–Whitney tests, SNH admissions for brain tumors overall had higher rates of inpatient mortality (*P* < .001) and complications (*P* < .001), but there were no differences in favorable discharge disposition or HACs (Supplementary Figure 2A–D). Overall LOS, postoperative LOS, and hospital costs at SNHs were elevated for every tumor subtype (all *P* < .001; Supplementary Figure 2E–G).

### Adjusted Differences in Inpatient Outcomes at SNHs

**Figure 2.**
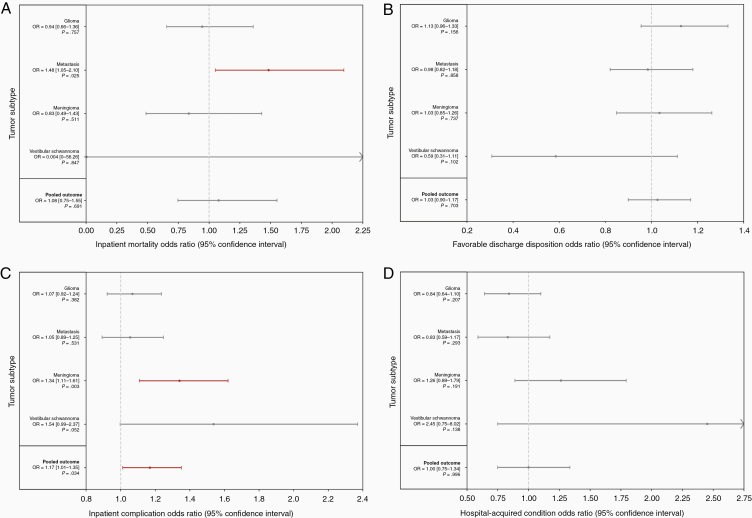
Inpatient mortality, discharge disposition, and complications. multivariate logistic regression was used to calculate adjusted odds ratios. (A) Association between safety-net status and inpatient mortality. (B) Association between safety-net status and favorable discharge disposition status. (C) Association between safety-net status and odds of experiencing an inpatient complication. (D) Association between safety-net status and odds of experiencing a hospital-acquired condition.

Following multivariate adjustment for patient, severity, and hospital characteristics, surgery at SNHs was associated with increased mortality for only metastases (OR = 1.48, *P* = .025; Figure 2A) but no other subtype. There were no differences in favorable discharge disposition rates (Figure 2B). Additionally, meningioma admissions exhibited higher complication rates following multivariate adjustment (OR = 1.34, *P* = .003; Figure 2C). A pooled analysis also demonstrated greater complications for the whole tumor population at SNHs (OR = 1.17, *P* = .034). However, there were no differences in HACs, a more limited cohort of in-hospital adverse events, at SNHs (Figure 2D).

**Figure 3.**
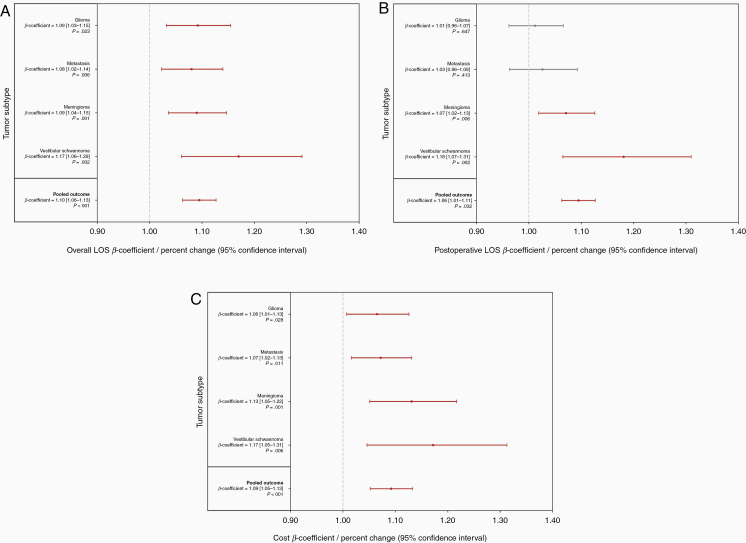
Length of stay (LOS) and hospital costs. Multivariate gamma log-link regression was used to calculate adjusted β-coefficients, corresponding to percent changes in LOS or costs. (A) Association between safety-net status and overall LOS. (B) Association between safety-net status and postoperative LOS. (C) Association between safety-net status and hospital costs.

All four tumor subtypes exhibited greater overall LOS at SNHs, a 10% elevation in LOS at SNHs after multivariate adjustment (β-coefficient = 1.10, *P* < .001) or approximately +0.7 days overall (Figure 3A). Meningiomas, VSs, and the overall population had an increased postoperative LOS (β-coefficient = 1.06, *P* = .032; Figure 3B). SNH admissions for all four tumor subtypes had elevated adjusted inpatient costs, ranging from 6% more for gliomas (β-coefficient = 1.06, *P* = .028) to 17% more for VS (β-coefficient = 1.17, *P* = .006; Figure 3C). For brain tumors overall, safety-net status was associated with a 9% increase in costs (β-coefficient = 1.09, *P* < .001) or roughly $2,292 more per admission. There was no heterogeneity for any outcome (all *P* > .10).

To adjust for the potential influence of in-hospital events on LOS and inpatient costs, we performed a sensitivity analysis, whereby multivariate regression for LOS and costs was repeated with complications and HACs added as two additional confounders (Supplementary Figure 3). LOS remained higher for all tumor subtypes and the overall population (β-coefficient = 1.07, *P* < .001). Expenses were greater for all subtypes—except glioma—and brain tumor craniotomies overall (β-coefficient = 1.08, *P* < .001).

Finally, we assessed differences between SNHs classified as high volume (top 20% in terms of brain tumor craniotomy volume) or low volume (bottom 80%). There were insufficient VS admissions for analysis. Low-volume and high-volume SNHs did not have any differences in mortality, discharge disposition, or complications (Supplementary Figure 4). High-volume SNHs exhibited higher odds of HACs for meningioma admissions (OR = 2.13, *P* = .036) compared to low-volume SNHs. However, for the overall study population, high-volume SNHs had lower overall LOS (β-coefficient = 0.90, *P* = .013) and postoperative LOS (β-coefficient = 0.90, *P* = .021) compared to low-volume SNH. There were no differences in costs.

## Discussion

SNHs serve a disproportionately vulnerable patient population. SNH stability is important for patients of all backgrounds, since closure of SNHs may cause spillover of undercompensated care to neighboring facilities, challenging hospitals of all levels.^26^ We found that brain tumor craniotomy SNH patients were more likely to be non-white, uninsured, and low income (Table 3), which are factors that earlier studies have correlated with decreased access, adverse discharge disposition, and excess mortality.^2,5,27^ Poorer outcomes in vulnerable patients may be attributed to higher rates of comorbidities and barriers like health literacy, social support, and access to preventive care. The greater frequency of patients in our study population from the highest income quartile (29.0%), compared to the lowest (20.3%), may reflect the impaired medical access and shorter overall life expectancies documented in low-income geographies.^2,28^ While uninsured rates have decreased over the past decade in many states, the share of inpatient Medicaid admissions grew by over 60% from 2000 to 2015, emphasizing the growing importance of this vulnerable population within inpatient care.^29^ Our study shows that treatment at SNHs, which disproportionately serve this segment of the population, may influence outcomes in the setting of inpatient brain tumor surgery.

While SNH status was unassociated with differences in discharge destination or HAC rates, SNHs exhibited higher mortality rates for metastases as well as elevated complications for meningiomas and the overall study population. Several mechanisms could explain this. SNHs may serve patients with higher disease severity compared to their non-SNH counterparts. For brain tumor craniotomies, SNH patients were more likely to be admitted nonelectively and present with higher severity of illness and risk of mortality scores. Certain subtype-specific variables were less optimal at SNHs, including higher rates of lung cancer and hydrocephalus for metastasis and VS patients, respectively. Prior studies have reported larger or more severe lesions at presentation for glioblastoma and pituitary adenoma patients at SNHs.^11,12^ Higher brain tumor severity of illness at presentation may be the byproduct of barriers that limit access to care.^30^ Because certain outcome differences did not persist following multivariate adjustment for severity metrics, such as increased mortality for glioma, greater presentation severity may partially account for observed outcome differences.

Similar to Brandel and colleagues’ analysis of glioblastoma patients, our study found that SNHs have lower annual brain tumors case volumes.^11^ Improvements in physician experience, coordination, and care processes conferred by higher hospital case volume may in part explain suboptimal outcomes at SNHs with lower caseloads. These factors may be especially critical for brain tumors outcomes due to the complex, multidisciplinary nature of treatment.^11^ However, the small differences in mortality and complications found in our study between low-volume and high-volume SNHs suggest that other factors beyond caseload may contribute to disparities. Resource limitations at SNHs may impact their ability to invest in quality improvement processes or to adopt newer technologies and treatment modalities, which may be more widely available at non-SNHs.^11^ These resource constraints may also help explain the lower rates of tumor resection for glioma patients at SNHs (93.3%), compared to non-SNH patients (94.2%). Discrepancies in operative management may also be due to SNH patients being more likely to be ruled out as surgical candidates because of higher severity on presentation,^11,12,31^ or lower trust in the healthcare system among marginalized patients due to past negative interactions and historical patterns of discrimination.^32,33^ Alternatively, patients with brain tumors may have been transferred from a SNH to non-SNH for their surgery, reducing resection rates at SNHs; surgeons in non-SNHs may also have varying incentives, such as different compensation arrangements, that lead to higher rates of resection. However, the NIS does not track this type of information, which may be a useful line of inquiry in future studies.

We found that poorer outcomes were especially concentrated among metastasis, meningioma, and VS admissions. A potential explanation is the more prevalent use of stereotactic radiosurgery (SRS) as upfront treatment for these three subtypes, relative to gliomas. Consequently, we hypothesize that surgical candidates for metastasis, meningioma, or VS at SNHs may encompass patients with large, complex lesions that were not SRS candidates; our multivariate models cannot adjust adequately for this increased case complexity. Earlier research has demonstrated disparities in the surgical treatment of these subtypes; one decade-long NIS analysis found that African Americans were nine times more likely to die, compared to Caucasians, following surgical excision of VS.^31^ While the NIS lacks more granular tumor data to substantiate this hypothesis, our results suggest that different tumor subtypes may have unique outcome patterns following surgery at SNHs.

While this study used the NIS to examine SNH brain tumor outcomes that have not been characterized nationally to date, including complications, the database only contains information on the index admission. We could not analyze long-term outcomes, use of adjuvant therapy, long-term postresection survival, rehospitalization, and reoperation. These outcomes have been examined in a small number of patients with glioblastoma, in which SNH patients had lower rates of adjuvant therapy and reduced overall survival.^11^ Interestingly, Brandel et al. found that differences in long-term survival no longer persisted if treatment differences were controlled, a finding that was corroborated by another single-institution study.^11,34^ Thus, an influential driver for disparities in long-term glioblastoma outcomes may be more limited access to adjuvant therapy and the range of care options at SNHs. However, our study highlights that there may also be differences at the inpatient level that may need to be targeted to improve care for brain tumor patients treated at SNHs.

In contrast to the differences across subtypes observed for mortality and complications, resource use, and expense were consistently elevated at SNHs across all four subtypes. This parallels findings by Hoehn et al., demonstrating higher LOS and costs in seven of nine general surgery and orthopedic procedures.^19^ However, another analysis of surgical inpatient admissions as a whole demonstrated lower mean LOS and costs at SNHs, suggesting potential variation in SNH outcomes between surgical subspecialties.^6^ These differences persisted after adjusting for complications and HACs; differences in LOS and costs for SNH patients may not be driven solely by adverse events during admission. Earlier studies suggested that lengthier hospitalizations for SNH patients may be due to inefficient care processes and constrained coordination of care, with less access to post-hospital rehabilitation or support for underinsured or uninsured patients.^7,19,35^ Infrastructure at higher volume institutions with more resources, such as enhanced care pathways and social services, may explain why high-volume SNHs had reduced LOS compared to low-volume SNHs. While the increased costs observed for brain tumor admissions at SNHs may be a natural consequence of prolonged LOS, inefficient care and coordination may raise the cost of treatment at SNHs.^7^ Poorer performance metrics may place additional financial penalties on SNHs via performance-based reimbursement models like value-based purchasing and the Hospital Readmissions Reduction Program.^35–38^ Because hospital financial health is associated with outcomes, these reimbursement penalties may exacerbate disparities for brain tumor care by placing more financial constraints on these fragile systems.^38–41^

Our findings suggest several policy considerations. Go et al. questioned whether poorer outcomes warrant the diversion of patients away from SNHs to more experienced institutions for certain procedures.^40^ Reimbursement models may facilitate centralization of high-risk or high-cost procedures at institutions that meet certain volume or quality thresholds.^42,43^ Nevertheless, these measures must also address critical challenges, including the resources needed to create referral networks and exacerbation of the already-high travel times for vulnerable patients.^11^ Alternative interventions may seek to stabilize the financial health of SNHs. One method is improving policies adjusting for a patient’s severity of illness and social challenges when reimbursing based on performance.^19,36,44^ Other studies suggested realigning quality and reimbursement links to reflect clinical priorities of SNHs, more robustly, as opposed to areas like patient experience scores, which may be impacted by socioeconomic factors.^35–37^ Further reimbursement reforms include assessing performance improvements over time, benchmarking SNH outcomes to peer institutions, and rewarding the achievement of equitable or superior outcomes.^19,35,37^ Addressing resource limitations at SNHs may not only ameliorate the deficiency of services that are known contributors to outcome disparities, such as adjuvant therapy, but also limit cutbacks on less profitable hospital services addressing social maladies among vulnerable patients, such as hospital-based preventive programs and post-hospitalization rehabilitation, which may improve outcomes like LOS.^19^ An important area to study is elucidating the drivers of poorer inpatient outcomes among SNH patients to target these disparities.

Finally, although our study was not able to assess brain tumor outcomes at SNHs post-2011 due to changes in the sampling methodology of the NIS, the more recent impacts of the Patient Protection and Affordable Care Act (ACA) on safety-net care merit discussion. Provisions in the ACA, including the establishment of health insurance marketplaces and particularly the expansion of Medicaid in nearly 40 states, increased insurance coverage by nearly 18 million from 2013 to 2016,^45^ which has been demonstrated to increase patient access to and early presentation of care for cancer and surgical conditions.^46,47^ Moreover, SNHs had increased patient volume and revenues in states that expanded Medicaid, suggesting continued demand for care at these institutions, while the opposite was observed in nonexpansion states.^48–50^ Lindrooth et al. also determined that Medicaid expansion was associated with reduced hospital closures, particularly for institutions disproportionately serving uninsured patients.^51^ These trends collectively suggest that improved coverage for safety-net patients as well as improved volume and resources at SNHs may have improved SNH outcomes following the passage of the ACA and partially ameliorated the disparities documented in this study, but this has yet to be validated in the setting of brain tumor surgery. Nevertheless, uninsured rates have increased since 2017 due to factors like the elimination of the individual mandate, continued gaps in coverage in Medicaid nonexpansion states, and the passage of Medicaid work requirements in certain states.^26^ Moreover, decreases in Disproportionate Share Payments to SNHs, which were planned due to expected decreases in uninsured patients but have not been adjusted for factors like Medicaid nonexpansion, and high penalizations for SNHs through value-based reimbursement models introduced by the ACA may exacerbate financial constraints at these institutions.^9,35^ In summation, the multifaceted impact of the ACA on safety-net care for brain tumor surgery warrants further research.

### Limitations

This study has limitations. First, coding errors in national administrative databases may occur, which may influence coding of certain outcomes, including complications.^52,53^ Notably, data abstraction for ICD-9 diagnosis codes in the NIS is performed after the patient’s admission, and these codes do not reflect whether the patient’s specific diagnosis was known before their operation, such as if their tumor was a glioma or metastasis. However, our exclusion of patients with ICD-9 codes for more than one tumor subtype may have reduced the inclusion of patients with multiple distinct malignancies or suspected diagnoses that may have complicated management. Second, the NIS has limited cancer-specific variables, such as baseline Karnofsky Performance Status and extent of resection. Consequently, the necessity of using ICD-9 codes to generate severity metrics like malignant status in this study may leave residual confounding. Third, NIS does not report postadmission data like reoperation and readmission. Fourth, as discussed earlier, provisions in the ACA may have changed brain tumor outcomes at these institutions since 2011, the endpoint of our study period. Data beyond 2012 utilized a different sampling methodology that does not include all admissions for each individual hospital, making accurate calculations of safety-net burden impossible. Finally, due to limitations inherent to NIS, this study presents an incomplete picture of the broad SDoH impacting outcomes, such as language and housing stability.^44^ Nonetheless, our study represents a nationally generalizable analysis of how hospital safety-net status may influence neurosurgical outcomes in patients undergoing brain tumor surgery.

In conclusion, among 304,719 patients undergoing craniotomy for brain tumor, SNH patients exhibited poorer outcomes including increased in-hospital mortality for metastasis admissions, higher complication rates, and greater LOS and hospital costs across all subtypes. Further research into the causes of these disparities and interventions to rectify poorer SNH outcomes is warranted.

## Supplementary Material

vdaa167_suppl_Supplementary_Figures

vdaa167_suppl_Supplementary_Tables_S1-S2

## Notes

**Authorship Statement.** Conceived and designed the study: O.Y.T., K.M.R.P., and S.A.T. Analyzed the data: O.Y.T., R.K.L., R.J.W., S.A.T. Wrote the paper: O.Y.T., K.M.R.P., R.J.W., and S.A.T. Critically revised the manuscript: All authors. Reviewed and approved the final manuscript: All authors.
