# Supplementary material for: The impact of hospital safety-net status on inpatient outcomes for brain tumor craniotomy: a 10-year nationwide analysis
Source: Neurooncol Adv. 2020 Dec 1;3(1):vdaa167. doi: 10.1093/noajnl/vdaa167 (PMC7813162; doi:10.1093/noajnl/vdaa167)
Supplement: vdaa167_suppl_Supplementary_Figures [file vdaa167_suppl_supplementary_figures.docx]

**Supplementary Figures:**

**Supplementary Figure 1: Study Population Flow Diagram of Inclusions and Exclusions.** Flow diagram visualizing selection of the study population, nationwide admissions for brain tumor craniotomy from 2002-2011, used in this analysis.


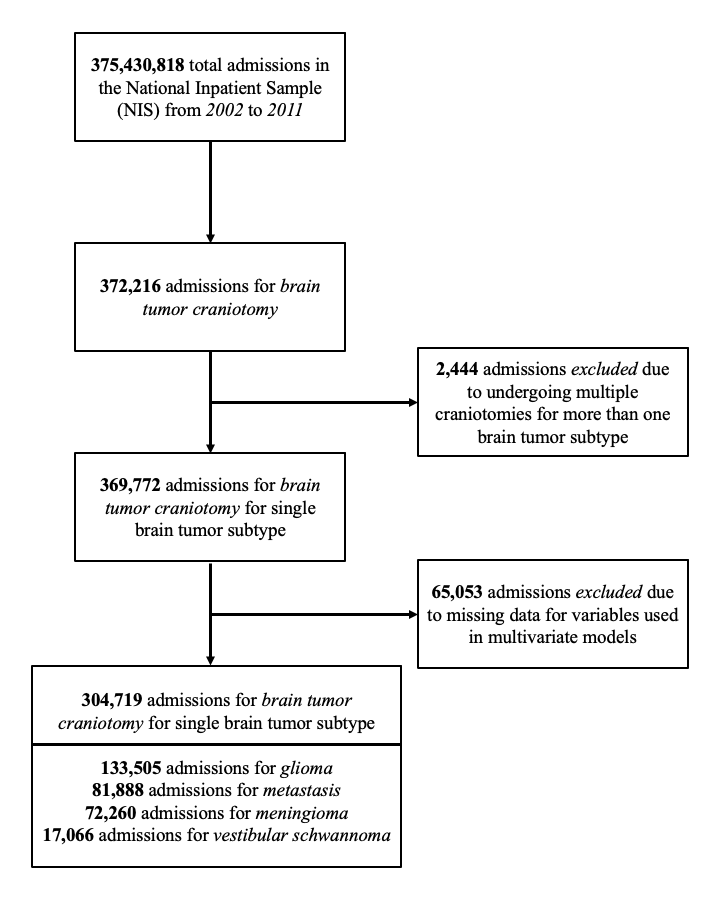


**Supplementary Figure 2: Unadjusted Differences in Inpatient Outcomes.** Univariate logistic regression and Kruskal-Wallis hypothesis tests were used to compare unadjusted differences in binary and continuous outcomes, respectively, between non-SNH and SNH admissions. **A:** Unadjusted difference in inpatient mortality rates. **B:** Unadjusted difference in favorable discharge disposition status rates. **C:** Unadjusted difference in inpatient complication rates. **D:** Unadjusted difference in HAC rates. **E:** Unadjusted difference in overall LOS. **F:** Unadjusted difference in postoperative LOS. **G:** Unadjusted difference in hospital costs.





**Supplementary Figure 3: Sensitivity Analysis for LOS and Hospital Costs.** Development of an inpatient complication and development of an HAC were added as additional confounders to multivariate regression for LOS and hospital costs. **A:** Association between safety-net status and LOS. **B:** Association between safety-net status and hospital costs.


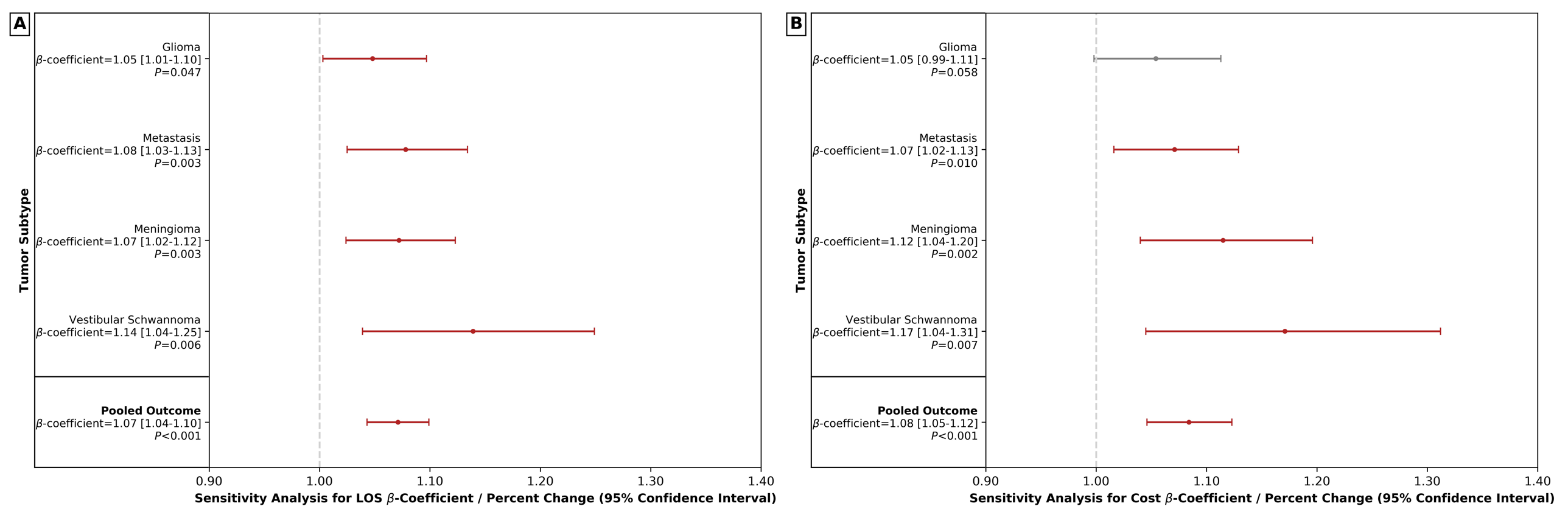


**Supplementary Figure 4: Adjusted Differences in Outcomes Between Low-Volume and High-Volume SNHs.** Multivariate regression was conducted exclusively for SNH admissions to determine the association between admission to a high-volume SNH (vs. a low-volume SNH) and inpatient outcomes. VS did not have enough admissions for analysis. **A:** Association between high-volume SNH treatment and inpatient mortality. **B:** Association between high-volume SNH treatment and favorable discharge disposition status. **C:** Association between high-volume SNH treatment and experiencing an inpatient complication. **D:** Association between high-volume SNH treatment and experiencing an HAC. **E:** Association between high-volume SNH treatment and overall LOS. **F:** Association between high-volume SNH treatment and postoperative LOS **G:** Association between high-volume SNH treatment and hospital costs.

Postop. = postoperative.
