# Supplementary material for: The impact of hospital safety-net status on inpatient outcomes for brain tumor craniotomy: a 10-year nationwide analysis
Source: Neurooncol Adv. 2020 Dec 1;3(1):vdaa167. doi: 10.1093/noajnl/vdaa167 (PMC7813162; doi:10.1093/noajnl/vdaa167)
Supplement: vdaa167_suppl_Supplementary_Tables_S1-S2 [file vdaa167_suppl_supplementary_tables_s1-s2.docx]

**Supplementary Table 1: ICD-9 Codes for Identification of Inpatient Complications**

| **Complication Subtype** | **ICD-9 Codes** |
| --- | --- |
| Cardiovascular | 458.2, 458.29, 997.1, 997.2, 997.71-997.79, 999.2, 453.4, 453.41-453.49, 451.19, 451.2, 451.81, 451.11 |
| Device | 349.1, 996.2, 996.4, 996.40-996.49, 996.59, 996.6, 996.6-996.69, 996.7, 996.70-996.79 |
| Gastrointestinal | 564.4, 579.3, 997.4, 997.41, 997.49 |
| Genitourinary | 997.5 |
| Hemorrhage or hematoma | 998.1, 998.11, 998.12, 998.13 |
| Infection | 998.5, 998.51, 998.59, 999.3, 999.31-999.39 |
| Neurologic | 997.0, 997.00-997.09 |
| Respiratory | 415.1, 415.11, 415.19, 512.1, 512.2, 518.7, 997.3, 997.31, 997.32, 997.39 |
| Serum/transfusion reaction | 276.61, 999.4, 999.41, 999.42, 999.49, 999.5, 999.51, 999.52, 999.59, 999.6, 999.60-999.69, 999.7, 999.70-999.79, 999.8, 999.80-999.89 |
| Shock | 995.4, 998.0, 998.00, 998.01, 998.02, 998.09 |
| Other | 277.83, 349.31, 780.62, 909.3, 995.24, 995.86, 997.9, 997.91, 997.99, 998.2, 998.3, 998.30-998.33, 998.4, 998.6, 998.7, 998.8, 998.81, 998.83, 998.89, 998.9, 999.1, 999.9 |

ICD-9 codes to identify inpatient complications were derived from previous NIS studies of neurosurgical procedures as well as validated Clinical Classifications Software algorithms, which are used to identify the most common inpatient medical and surgical complications. An admission was categorized as experiencing an inpatient complication if one of the aforementioned ICD-9 codes was recorded.

**Supplementary Table 2: Distribution of Primary Tumor Sites for Brain Metastasis Admissions**

| **Primary Tumor Site** | **Total Number (%) for Overall Metastasis Admissions** | **Total Number (%) for Metastasis Admissions at Non-SNHs** | **Total Number (%) for Metastasis Admissions at SNHs** | ***P*-Value** |
| --- | --- | --- | --- | --- |
| Lung | 27,782 (33.9%) | 24,157 (33.4%) | 3,625 (38.1%) | <0.001*** |
| Breast | 2,976 (3.6%) | 2,453 (3.4%) | 523 (5.5%) | <0.001*** |
| Skin | 2,112 (2.6%) | 1,898 (2.6%) | 214 (2.2%) | 0.413 |
| Kidney | 1,624 (2.0%) | 1,453 (2.0%) | 171 (1.8%) | 0.794 |
| Colon | 927 (1.1%) | 796 (1.1%) | 131 (1.4%) | 0.044*** |

The proportion of brain metastasis admissions with a tumor in the 5 most common primary tumor sites (lung, breast, skin, kidney, colon) were identified using ICD-9 codes. Nonparametric Mann-Whitney tests were used to detect significant differences in characteristics between admissions at SNHs compared to admissions at non-SNHs.
